# Reports of Surgical Cases

**Published:** 1872-05

**Authors:** F. A. Stanford

**Affiliations:** Columbus, Ga., Chairman of Committee on Surgery for the Third Congressional District of Georgia


ATLANTA

### MEDICAL AND SURGICAL JOURNAL.

VOL. X.—MAY, 1872.—NO. 2.

### ORIGINAL COMMUNICATIONS.

### REPORTS OF SURGICAL CASES.

BY F. A. STANFORD, M.D., COLUMBUS, GA.,

*Chairman of Committee on Surgery for the Third Congressional District of Georgia.*

[Presented to the Georgia Medical Association, at its Annual Meeting, in Columbus, 1872.]

*PLACENTA PRÆVIA—TREATMENT BY RUPTURE OF THE MEMBRANE, EVACUATION OF THE LIQUOR AMNII, AND SEPARATION OF THE PLACENTA FROM ITS UTERINE CONNECTIONS.*

Having, during the last few months, an opportunity of putting into practice the above treatment of placenta prævia, it gives me pleasure to contribute my experience to the history of this, the most dangerous of all the complications of labor. The particulars of the two cases I shall here describe, as circumstantially as a respect for brevity will admit, I hope will serve to convince this body, representing the intelligence of the profession of the State, of the wisdom of the practice insisted upon by that greatest of modern gynæcologists, Sir James Y. Simpson, whose light has gone from us, except in the imperishable record he has left behind.

Called, on the morning of the 8th of June, 1871, to attend Mrs. —— in her tenth confinement, I found her in active labor, and was informed by her that she was having profuse hemorrhage. About ten days previous to this, I was called to attend her in a sudden attack of hemorrhage; and as she had just passed the seventh month of her pregnancy, I was induced to suspect a placenta presentation. I advised, at this visit, that she remain close to her apartment, and to guard as much as possible against a return of hemorrhage. This advice had been strictly observed. Finding now that she was in labor, the pains being regular and with some force, I made no delay in satisfying myself of her true situation.

Upon examination, I found the os soft and relaxed, and dilated to about the size of somewhat more than a half inch in diameter. With the index finger moved within and upward, I detected that it was a placenta presentation. At this time, I introduced a sponge tampon well up, and in contact with the os.

Much alarmed at the magnitude of the case before me—occurring, too, as it did, in a frail and delicate subject, who could badly afford to lose much blood—I administered a full opiate, and hurried my carriage back to the city for professional assistance. Very soon, Dr. Mason was with me. In consultation, it was decided that we proceed to effect delivery by turning—the patient by this time having well reacted, and the uterine pains being active. Placing her in position for our greatest convenience, and removing the tampon, I attempted the dilatation of the os by the introduction of the index and middle finger, hoping to effect an entrance of the entire hand. In this I failed, being able alone to enter the two fingers. In accomplishing so little as this, the most alarming hemorrhage followed; the patient fainted away, and I, as promptly as possible, reintroduced the tampon, lowered her head, and gave her freely of brandy and opium. After an interval of about an hour, reaction being very well established, and the morale of our patient being most excellent, an effort was made by Dr. Mason to accomplish what I had failed to do. The Doctor failed, and the same exhaustion from loss of blood followed. By a discontinuance of farther effort ou our part, and by the free use of stimulants and nutrients, the good patient rallied again.

It was now apparent, to both Dr. Mason and myself, that our patient would succumb if we attempted again to accomplish a forcible delivery. We could not enter the womb through the small os without the greatest violence, nor could the patient survive the shock and loss of blood which would necessarily follow such a heroic undertaking; and yet we could not remain passive, as uterine contraction was active and the hemorrhage free. Under these very grave circumstances, it was decided that we would puncture the membrane, evacuate the water, and then separate the placenta from its uterine attachments. I now proceeded to carry this determination into practice. With a good-sized knitting-needle (previously having removed the tampon), guided by the finger, I had little difficulty in passing through the placental mass, and puncturing the membrane. This was soon made known by the flowing of the warm liquid over my hand, and into a napkin placed to receive it. I then, with the fingers, separated the placenta for very nearly its entire circumference. A small part I found morbidly adherent, which was left in place. Both hemorrhage and uterine pains ceased about this time. We employed a white linen handkerchief, to serve both as tampon and an index to tell us of hemorrhage, if such should occur. We made the patient comfortable, and gave her freely of stimulants and nourishment; and with instructions to her lady nurses to observe the linen closely—that they might detect any return of hemorrhage—left our patient to all the rest she could get.

At this stage, additional consultation, in the person of Dr. Samuel A. Billing, arrived. Informing him fully of what had occurred, and what was done, it was fully agreed to leave her to her rest.

The patient about this time fell into a good sleep, which lasted for about two hours, when she awoke much refreshed, cheerful and bright. Upon thorough inspection of the linen, not a particle of hemorrhage had taken place. She laughingly told us that we could now rest ourselves, and not disturb about her, as she would give us no trouble until about one o'clock the next morning; this was about two P.M.

She continued doing well, sleeping at intervals, and taking nourishment through the afternoon and evening, and very nearly about one o'clock in the morning, sent me the information that she was having pain. I was soon by her side, and found the labor progressing. Removing the tampon, I could discover no blood-stains. I found the os dilating, and the placental mass occupying it well, and protruding. The labor continued active, and at four o'clock A.M., she was delivered of a still-born male child. The placental mass, upon being in advance of the head at the commencement, was pushed to one side, permitting the passage of the child; and in attempting its delivery, I found the adhesion, before referred to, so firm as to require considerable force to pull it off, which was done by Dr. Mason whilst I made traction upon the mass.

Our patient did well, and is now the happy center of the husband and several interesting children.

Case No. II.—My second case of placenta prævia was not long in following the first. And just here I hope that I may be pardoned a little digression; how often do we notice a running together of extraordinary cases in medicine and in surgery, whether it be obstetric or general.

Lindsey (colored), on July 3d, 1871, requested me to visit his wife, then in labor, and mentioned that the midwife in attendance had informed him that everything was wrong.

But a few blocks distant, I was very soon with the patient, and found her flooding most profusely. She was already in suitable position—across the bed. Upon examination, a complete implantation of placenta was found over the os, and this was found dilated to an extent which would easily admit of the introduction of all of the fingers. Recognizing the situation, I sent the husband for assistance; but finding the hemorrhage so profuse, and the life of the patient so much endangered, I ruptured the membrane, evacuated the water, and separated the placenta from its attachments. Labor being very vigorous, the placenta was soon delivered in mass. Dr. Billing, who had been sent for, arrived just in time to receive this and separate the fœtus. Very soon a five months' fœtus, as a footling presentation, was delivered.

This patient had no hemorrhage after the rupture of the membranes and separation of the placenta.

P. S.—For a most interesting and exhaustive treatise upon this subject, I would most respectfully refer to the "Selected Obstetrical and Gynæcological Works of Sir James Y. Simpson, as published by the Messrs, Appleton, New York.

---

*PUERILE INCONTINENCE OF URINE—TREATMENT BY BOUGIE.*

In many of the medical journals, not a great while since, was published a treatment of this very annoying difficulty, as pursued by a distinguished Irish surgeon, Sir D. J. Corrigan. This treatment, most of you will remember, consisted in tying the prepuce before dismissing the young subject for sleep. Not finding it necessary to adopt the practice advised by so distinguished an author, I can not speak of its merits, but have found the above treatment (the introduction of the bougie) to answer the best of purposes in several cases in which I have employed it.

In 1865, after the close of the war, after we had returned to the peaceful pursuits of civil life, an extreme case of puerile incontinence sought me for treatment, in the person of a lad about fourteen years of age. He had been troubled all his life, since his earliest recollection, with an inability to retain his water longer than a few hours, day or night. During the day, whilst at school, permission was given him to leave the room when he desired; and at night, when in bed, watchers were required to be placed over him, so as to arouse him at certain intervals—otherwise he was sure to wet his bed. This case was a very melancholy one to me, and elicited much of my sympathy. After much inquiry, I ascertained that he had been through the ordinary routine of treatment usually practiced in similar cases, and under the care of able physicians. It was, therefore, very plain to me, that I could expect little from medicine in this case. Incontinence of urine, or at least a very frequent desire to evacuate the bladder, being one of the most prominent symptoms in pointing me to the existence of urethral stricture, and especially the desire being much greater through the late hours of night, and the marked relief of this difficulty afforded my patient from the very first introduction of the bougie into the bladder, determined me to try this method of treatment with my young friend. I was not disappointed; relief followed after introducing a number eight conical steel bougie. This being practiced daily for two weeks, the patient was discharged, considering himself cured. He continues well up to the present time.

Since the happy result that so signally followed the above case, I have had an opportunity of testing the practice in four other cases of boys, from the ages of four years up to nine, with the like result. All surgeons, with much experience in the treatment of urethral irritation, fully appreciate the benumbing effect of the introduction of the bougie upon the sensitive urethra, or an irritable bladder; and I most confidently recommend this treatment to their consideration. I have not yet had an opportunity of testing this course in a female, but see no reason why it should not be equally efficacious.

---

*A HYDATID MASS TAKEN FROM THE UTERUS.*

This very interesting specimen of hydatid was expelled from the uterus on the 15th of March last, in the instance of a married lady, who supposed herself to have reached the fourth month of pregnancy. A few days previous to the expulsion of the mass, on the 1st of March, she had her first symptoms of threatened miscarriage, at which time I was summoned to her, and found that she was losing pretty freely of blood. By resorting to the ordinary expedients for arresting hemorrhage, the discharge of blood was soon checked, and she made comfortable. On the 15th of March, as above mentioned, I was the second time called to attend her, and found the symptoms of miscarriage most decided. She was having strong uterine contractions, with profuse hemorrhage; this continued for several hours, when the mass here exhibited was expelled. This mass, at the time of its expulsion, was thoroughly examined, with a view of finding out whether or not a fœtus was contained—also everything which escaped from the uterus before and subsequent to its expulsion—but not a thing simulating such could be found.

Dr. Thomas, in his practical work on "The Diseases of the Womb," regards this form of hydatid as a degeneration of the chorion, and speaks of its exceeding rarity. Mr. William Tyler Smith, in his excellent work, furnishes a plate representing the same. His description, taken from other able authors, is very interesting—as, for instance, Meltenheimer considers the chorion as the center of the whole growth. "On the walls of this great vesicle a new generation of cysts is formed, and each of these cysts have the power of producing one or many daughter cysts; or, to use his precise words, 'Berry grows out of berry, and the stalks do not unite berries with the principal stems, but berries with berries, and lastly, with a central mother cyst.'"

extent of the surface of the tumor, about five inches, a large quantity of this fluid flowed out. Upon inspecting the interior of this sac, I found that I had before me a nest containing something. With my fingers passed in, I took from the nest, very easily, a tumor as large as a hen's egg. Passing the hand back, I withdrew another a little smaller; thence, repeating the process, another yet a little smaller; and thus the work went on until I removed from this nest twenty-one tumors, from the size of a hen's egg gradually down to a number five shot, when we ceased to count them.

After removal of these bodies, I examined into the condition of the parts which composed the nest of this collection. I found that it occupied all the space from the front to the outer aspect of the upper part of the arm, extending down upon the periosteum, which, however, was intact, the bone being not at all exposed. The wound was afterward treated by injections of carbolic acid, granulated, and healed very kindly from its depth to its surface. The young gentleman made a good recovery, and has experienced no difficulty in the use of the muscles of the arm. This being a specimen of hydatid tumor so unique and interesting in a pathological point of view, I presented it to my friend, Dr. William H. Van Buren, of New York city, who has it now in his collection.

---

### ABSTRACTS FROM GERMAN JOURNALS.

BY CH. RAUSHENBERG, M.D., ATLANTA, GEORGIA.

[Read Before the Atlanta Academy of Medicine.]

*ON THE TREATMENT OF VARIX BY SUBCUTANEOUS INJECTIONS OF ERGOTINE. By Dr. PAUL VOGT, Assistant Physician to the Surgical Polyclinic at the University of Greifswalde.*

After the observations of Von Langenbeck in relation to the cure of two aneurisms by subcutaneous injections of ergotine (*Berliner Klinische Wochenscrift*, 1869, No. 12, p. 117)—followed, first, by a communication of Schneider, made at the meeting of the Society for Scientic Medicine, at Koenigsberg, on the 25th

